# Effects of low-dose energy spectrum scanning combined with adaptive statistical iterative reconstruction on the quality of imaging in Budd-Chiari syndrome

**DOI:** 10.1371/journal.pone.0204797

**Published:** 2018-10-18

**Authors:** Lei Su, Liyang Chang, Qiang Sun, Lili Hu, Yan Wu, Jianbo Gao

**Affiliations:** 1 Department of Radiology, First Affiliated Hospital of Zhengzhou University, Zhengzhou, Henan Province, China; 2 Department of Stomatology, First Affiliated Hospital of Zhengzhou University, Zhengzhou, Henan Province, China; Brigham and Women's Faulkner Hospital, UNITED STATES

## Abstract

**Objective:**

To assess the quality and diagnostic accuracy of monochromatic images combined with adaptive statistical iterative reconstruction (ASIR) performed via spectral computed tomography (CT) in patients with Budd-Chiari syndrome (BCS).

**Methods:**

Sixty-two patients with BCS underwent pectral CT with upper abdominal two-phase contrast-enhanced scanning to generate a 60keV monochromatic energy level combined with ASIR (ranging from 0% -100%) during the portal venous phase (PVP) and the hepatic venous phase (HVP). One-way ANOVA was used to compare vessel-to-liver contrast-to-noise ratio (CNR) for the portal vein (PV), hepatic vein (HV), and inferior vena cava (IVC). Subjective evaluations of the images in the three groups were conducted by image quality assessors and compared via Kruskal-Wallis H test.

**Results:**

The CNR values of the PV trunk, HV, IVC, liver parenchyma and pancreas were within ASIR (ranging from 0% - 100%) weight, and the difference were statistically significant (*p* <0.05). The highest overall image score was distributed at 50% ASIR weight value. Higher CNR values of HV, hepatic parenchyma and pancreas were obtained in the IVC type than in mixed and HV types (respective *p* values = 0.035, 0.019 and 0.042). Higher CNR values of the IVC were obtained in the HV type than in mixed and IVC types (*p* = 0.032). The CNR value of the IVC in the mixed type was less than that of the HV type (*p* = 0.028). The CNR values of the HV and liver parenchyma in mixed type were lower than those of the IVC type (*p* = 0.016 and 0.038, respectively). The CNR value of pancreas in IVC type was higher than that of the HV type (*p* = 0.037). The diagnostic value of CNR in patients with the IVC type was higher than that in patients with mixed and HV type, while the diagnostic value of CNR was found to be the lowest for the HV type (*p* = 0.043).

**Conclusion:**

A monochromatic energy level of 60 keV with 50% ASIR can significantly improve image quality in cases of BCS.

## Introduction

Budd-Chiari syndrome (BCS) is characterized by obstruction in the hepatic venous outflow at the hepatic vein (HV) and the inferior vena cava (IVC). This results in the liver to heart reflux being affected thereby leading to a series of liver hemodynamic changes that manifest as a series of clinical symptoms[1]. Following the development of spectral computed tomography (CT), multi-parameter imaging has yielded abundant clinical diagnostic information. [2,3,4,5]. Currently, the application of spectral CT single-energy HV angiography predominantly pertains to portal vein(PV) angiography in patients with liver cirrhosis[6]. he present study of BCS CT angiography was limited to investigating the image quality of the best contrast-to-noise ratio (CNR) in single energy imaging for the PV, HV, and the IVC [7]. The primary aim of the study was to evaluate the quality of BCS angiography images of the spectral CT via single energy imaging combined with adaptive statistical iterative reconstruction(ASIR), and compare the image quality of different BCS types, in an effort to improve the quality of BCS angiography.

## Materials and methods

### Patients

The total of 62 BCS patients included in the study (41 men and 21 women, mean age 45.17 ± 11.13 years, age range 18–74 years) were diagnosed via surgery or Digital Subtraction Angiography(DSA) between May 2015 and September 2016. With regard to type distribution in the 62 cases, 11 were HV type, 7 were IVC type, and 44 were mixed (HV/IVC) type. The present study was approved by the Ethics Committee of The First Affiliated Hospital of Zhengzhou University, and all patients provided informed written consent before examination.

### CT examination

Unenhanced and two-phase contrast-enhanced CT examinations were performed using the Discovery CT 750 system (GE Healthcare, Wisconsin, USA) in a craniocaudal direction in spectral imaging mode, with an individual contrast agent, a dual flow rate of injection of contrast medium, and utilization of the portal vein trigger scan mode. After spectral CT, all patients underwent unenhanced imaging in a conventional helical mode at a tube voltage of 140kVp. Patients were then injected with non-ionic contrast medium (Iohexol Injection, Optiray 350, GE Healthcare through antecubital venous access at a rate of 2–4 mL/s (4 mL/s for total of I80% and then 2 mL/s for total of 20% and 2 mL/s for 40 mL saline) for a total of 80–140 mL (1.6 mL/kg) during the portal venous phase (PVP) and the hepatic venous phase (HVP). The imaging delay for PVP imaging was determined by automated image-triggering software (Smart Prep, GE Healthcare). PVP imaging was automatically begun 30 s after the trigger attenuation threshold (120 Hounsfield units) was reached at the level of the PV trunk and its branches. HVP imaging began 45 s after PVP imaging. PVP and HVP acquisitions were performed in the spectral imaging mode with a single tube. Other imaging parameters were slice thickness—5 mm, slice interval 5 mm, automatic tube current, rotation speed (gantry rotation time) - 0.8 s and helical pitch—0.984:1.

### Image analyses

**Fig 1:**
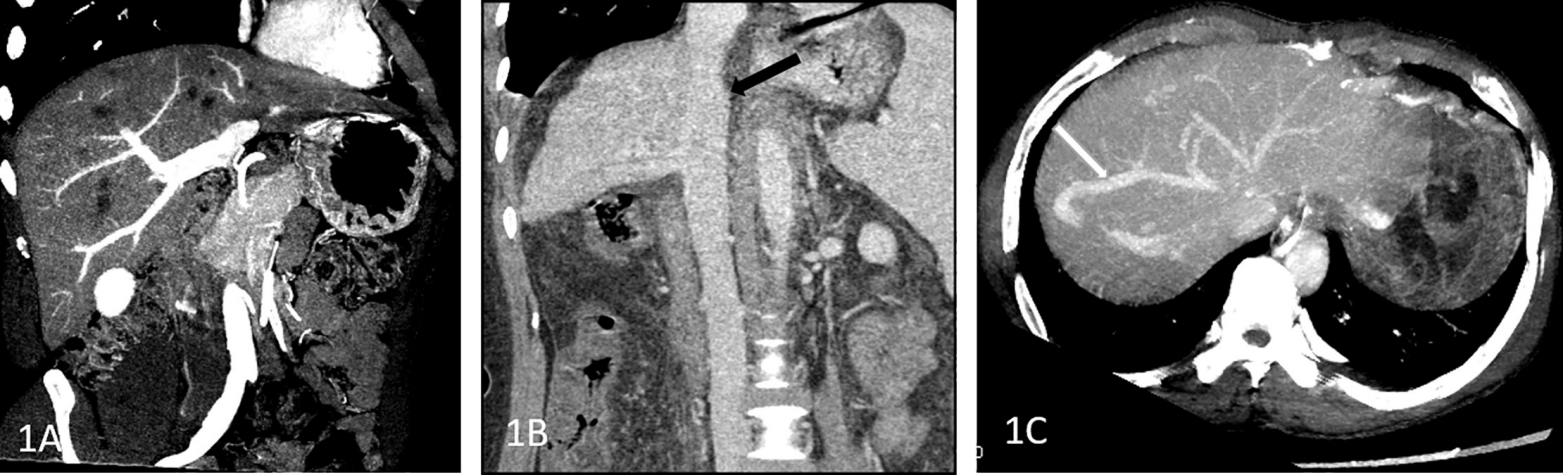
Vascular reconstruction of PV, HV, and IVC. A maximum intensity projection (MIP) image of the PV, showing the PV trunk and its branches clearly; Fig 1B curve planar reformation (CPR) image of the IVC, showing the IVC patency (black arrow); Fig 1C maximum intensity projection(MIP) image of the HV, showing the left HV, HV and right HV occlusion, subhepatic vein open, through the auxiliary hepatic vein (white arrow) into IVC.

The single energy images were derived from spectral CT (slice thickness 1.25 mm) combined with ASIR (from 0 to 100%). The reconstructed images were processed with GSI Volume Viewer software and an AW 4.6 workstation (GE HealthCare) to measure and record relevant parameters. Comprehensive post-processing technology is used to display vascular diseases clearly and intuitively. (Fig 1).

For images at each energy level with ASIR, the mean CT-derived values (in Hounsfield units) of vessels (*i*.*e*., PV, HV, and IVC) were assessed by manually placing circular or ovoid ROIs, which were drawn at the centrally within the segment (mean pixel number 57, range 34–85) from three contiguous slices. Mean CT data for the hepatic parenchyma and pancreas were obtained by manually placing circular ROIs at the same slice (mean pixel number 280, range 210–375). In cases where they were present, areas of focal changes in parenchymal density and large vessels were carefully avoided. A global assessment of image noise, defined as the standard deviation of the pixel values from a circular or ovoid ROI (mean pixel number 120, range 70–170) drawn in a homogeneous region of the subcutaneous fat of the anterior abdominal wall, was performed. To ensure consistency, all measurements were repeated three times at the three contiguous imaging levels, and average values were calculated. For all measurements the size, shape, and position of the ROIs were kept consistent between the two phases by applying the copy-and-paste function.

The lesion-to-liver CNR was calculated via the following equation: CNR = (CT_1_—CT_2_) / SD_2_. CT_1_ for the CT value of the blood vessel, hepatic parenchyma and pancreas, CT_2_ for the CT value of the subcutaneous fat, SD_2_ for the standard deviation of the subcutaneous fat.

### Subjective evaluation standard for image quality

Readers recorded the number of lesions, lesion size (defined as the maximum diameter measured with an electronic ruler on transverse images), and segmental location according to the anatomical segmentation schemes of Couinaud and Bismuth on a standardized template. For overall image quality [8], the scoring scale was as follows: 5 = no obvious image noise or artifacts, sharp anatomical structure and satisfactory detail; 4 = mild image noise and artifacts, less clear anatomical structure and detail; 3 = moderate image noise and artifacts, reduced confidence in details but anatomical structure still relatively clear; 2 = severe image noise and artifacts, confidence in details and anatomical structure reduced, diagnosis questionable; and 1 = severe image noise and artifacts, non-diagnostic.

With regard to BCS, a different subjective scoring system incorporating diagnostic confidence was evaluated. Scoring criteria are [9]: 1 = unclear, cannot be diagnosed; 2 = Half clear, diagnostic confidence <50%; 3 = Clear, diagnostic confidence 50% to 90% - 3 points; 4 = Very clear, diagnostic confidence> 90%.

### Statistical analysis

The SPSS 21.0 software package (IBM Corporation, Armonk, NY, USA) was used for statistical analysis. Normality of distribution variance homogeneity tests were applied to the data. The choice of ASIR weight value at a 60 keV monochromatic energy level energy level and the variation in in image quality of different BSC types were analyzed via one-way ANOVA. The Kruskal-Wallis H test was used to assess the overall image quality scores of the three groups of images. *p* <0.05 was deemed to indicate statistical significance.

## Results and discussion

### Comparison of CNRs in the PV trunk, HV, IVC, hepatic parenchyma and pancreas at different ASIR weight values

**Fig 2:**
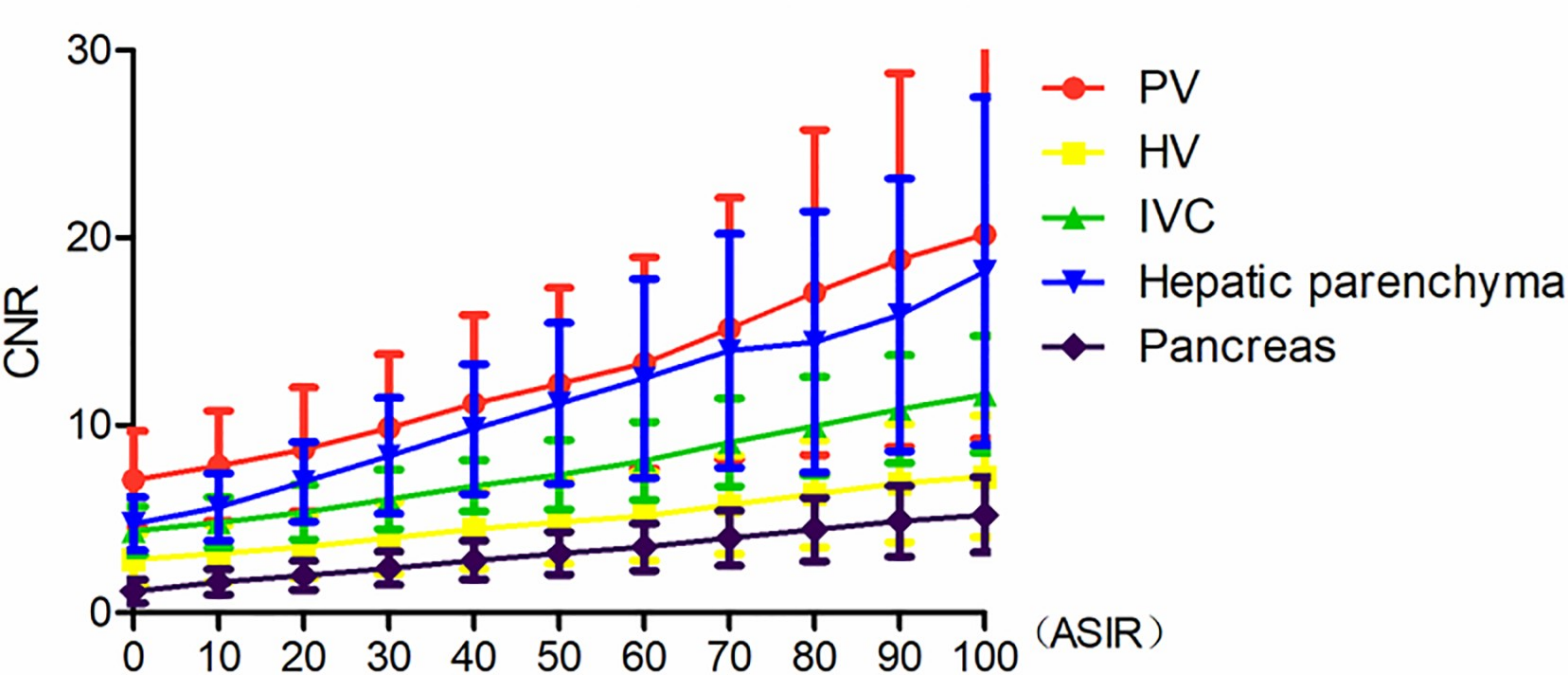
CNR values in the PV trunk, HV, IVC, hepatic parenchyma, and pancreas at 60keV. As the weight value of ASIR increased, the CNR value gradually increased.

CNR tended to increase with the increase in ASIR weight in the range of 0–100% ASIR weight at a 60 keV monochromatic energy level. CNR values were lowest at 0% ASIR, and highest at 100% ASIR. The difference in the PV trunk, HV, IVC, hepatic parenchyma, and pancreas CNRs from 0% to 100% ASIR weight was statistically significant (*p* < 0.05) (Fig 2).

### Subjective scoring of different ASIR weight values

**Fig 3:**
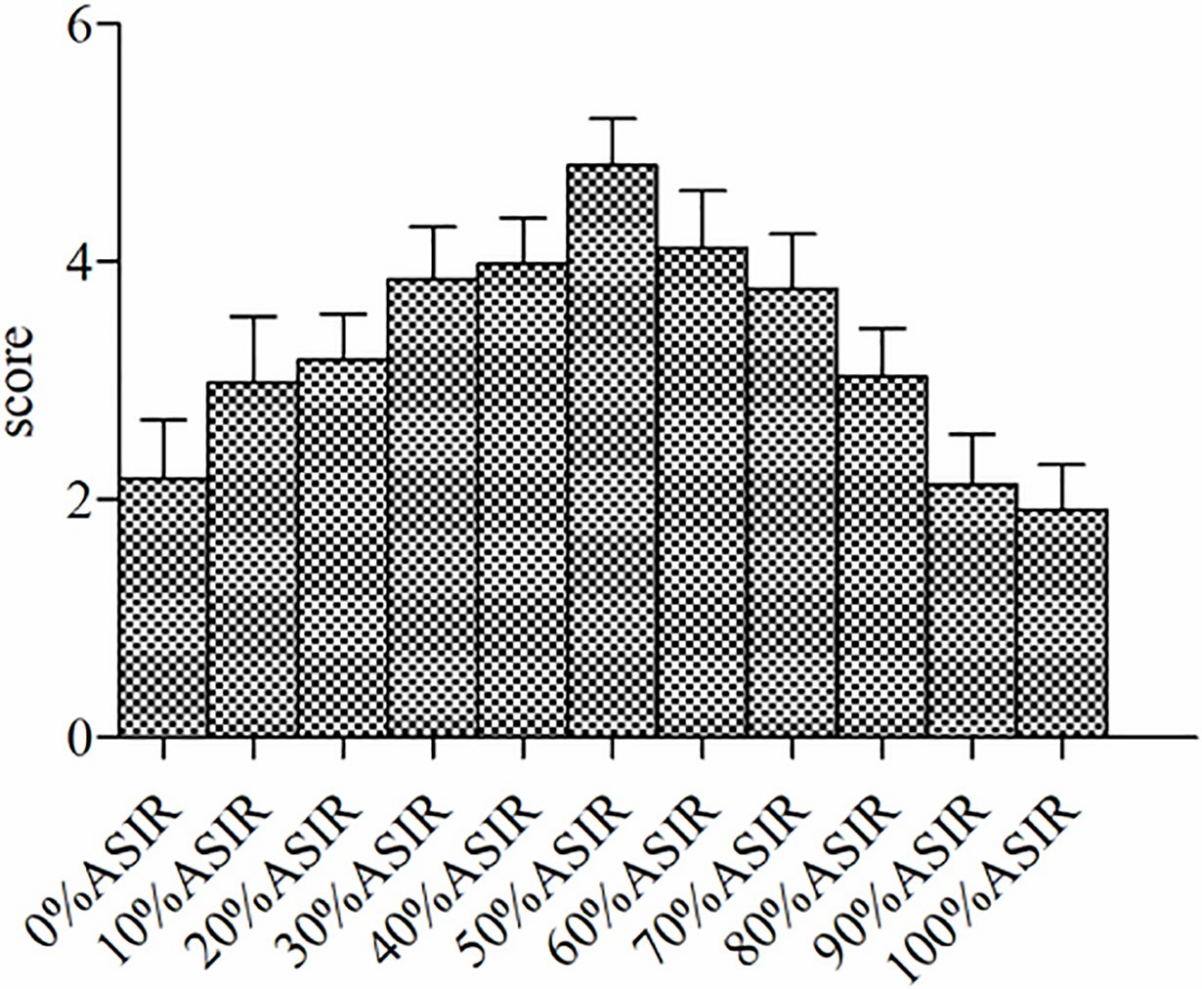
Overall image quality subjective with ASIR weight value curve. Fig 3 shows that the image quality of the ASIR weight value from 0% to 50% increases, and 50% to 100% decreases.

The image quality of the ASIR weight value from 0% to 50% increases, and 50% to 100% decreases, the image quality does not improve with the increasing ASIR weight value. The highest overall image score is at 50%. **(**Fig 3**)**

### Comparison of CNR values in different BCS types

**Fig 4:**
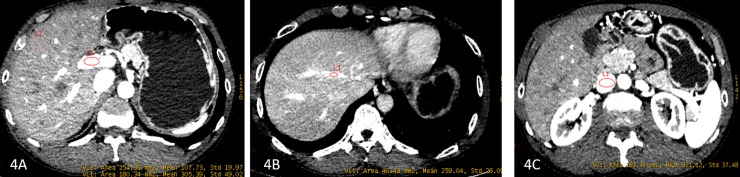
The CNR values of PV trunk, HV, IVC and hepatic parenchyma. Female, 57 years old, imaging diagnosis of BCS (HV type).Fig 4A–4C, Axial images, showed PV trunk, HV and IVC. The CNR values of PV trunk, HV, IVC and hepatic parenchyma were: 12.02, 4.09, 4.89, 5.64 and 1.61 respectively.

CNR values of the PV trunk, HV, hepatic parenchyma and pancreas in the IVC type were significantly higher than those in the mixed type and HV type, and with the exception of the PV trunk, these were statistically significant. The CNR of the IVC in the HV type was greater than that of the mixed type and the IVC type and the difference was statistically significant (*p*<0.05). The CNR of the IVC in the mixed type was lower than that of the HV type (*p* = 0.028, *p*<0.05). The CNR of the HV and hepatic parenchyma in the mixed type was lower than that in the IVC type (*p* = 0.016 and 0.038, *p* <0.05), and the CNR of pancreas in the IVC type was higher than that in the HV type, (*p* = 0.037, *p* <0.05). (Table 1, Fig 4)

**Table 1:** Comparison of CNR values for the PV trunk, HV, IVC, hepatic parenchyma and pancreas in different BCS types (mean ± SD).

Type	Cases	PV trunk	HV	IVC	Hepatic parenchyma	Pancreas
Mixed	44	13.74±3.76	4.40±1.51	3.64±1.59	5.83±1.49	2.09±1.05
HV	11	12.35±3.15	4.13±1.22	5.01±1.18	5.70±1.21	1.57±0.58
IVC	7	14.73±3.13	6.26±1.63	3.72±1.29	7.42±1.90	2.82±1.13
*F Value*		1.042	4.230	3.655	3.542	3,351
*P Value*		0.359	0.019	0.032	0.035	0.042

CNR, contrast-to-noise ratio; PV, portal vein; HV, hepatic vein; IVC, inferior vena cava; BCS, Budd-Chiari syndrome; SD, standard deviation. *p* < 0.05 was considered statistically significant.

### Diagnostic confidence subjective score for different types of BCS

Diagnostic confidence was highest for the IVC type and lowest for the HV type. The difference was statistically significant (*p* = 0.043), (Table 2).

**Table 2:** Diagnostic confidence subjective score for different types of BCS.

Group	Score of 1	Score of 2	Score of 3	Score of 4	Total	*x*^*2*^	*p* *Value*
Mixed type	0	9	23	12	44		
HV type	0	4	7	0	11	6.310	0.043
IVC type	0	0	4	3	7		

*p* <0.05 on behalf of the difference was statistically significant.

Spectral CT scaning can simultaneously yield mixed energy and monochromatic-energy images (40–140 keV) [10,11,12,13]. In general, the low energy levels with high contrast and high noise. Reconstruction algorithms are one the important factors that affect image quality. ASIR is an iterative reconstruction algorithm of the data based on the noise reduction model. A reduction in the radiation dose also helps reduce the noise as has been previously demonstrated[13,14,15]. The adaptive method is based on a complete understanding of the noise properties and correctingsuppressing them to get achieve clearer image.

Theoretically, the higher the ASIR weight value, the stronger the noise reduction capability. However, if the ASIR weight value increases beyond a certain point (say, approximately 80% ASIR), then the contrast in the image decreases significantly[16]. When the ASIR increases to more than 60%, the spatial resolution begins to decline as reported by Singh *et al* [17]. Therefore in order to maintain the spatial resolution of the image in an abdominal scan, the ASIR weight value should not exceed 60%. Studies have shown that the quality of chest and abdomen scan images decreases with increases in an ASIR weight value and so it is best to maintain ASIR weight value between 40%-60% [5,18].

In the current study, the ASIR weight value of the single energy image at a 60 keV monochromatic energy level was set from 0% to 100%, and the noise reduction rate of the image gradually increased with increasing ASIR weight value. At 0% ASIR the image noise reduction rate was the lowest, and at 100% it was the highest. CNR values gradually increased with increasing ASIR weight. CNR values were found to gradually increase with the increase in ASIR weight values. Subjective scoring results showed a decrease in score as ASIR increased beyond a point. When image noise was large, the score was low with ASIR weight value ranging from 0% to 20%. The spatial resolution began to reduce with ASIR weight value ranging from 70% -100%, and the image quality as well as the score was low with a "fuzzy" effect apparent in the image. Image quality acceptable at 30%-60% ASIR. The overall image score was the highest at 50% weight value. With higher ASIR value reconstruction, the spatial resolution of the image gets reduced and the noise value of the image changes leading to image distortion that cannot meet diagnostic requirements.

BCS types differ with regard to pathophysiological basis, clinical manifestation, clinical treatment, and prognosis [19,20]. Based on whether there were HV or IVC pathological changes, BCS was divided into HV type, IVC type and mixed type in the current study. The CNR values (except IVC) of the IVC type were greater than those of the HV type and mixed types. This is because the lesions simply involve the IVC and the obstruction of blood flow in the IVC, the reduction of blood flow rate and even reverse, which leads to lower inferior vena cava enhancement with relatively lower CNR. The CNR value of the IVC in the HV type was significantly greater than those of the mixed type and the HV type. This may be related to the simple involvement of hepatic veins, and IVC patency, which may result in the effect of IVC vascular enhancement being less. When we assess the diagnostic value of CT images, the diagnostic confidence of patients with the IVC type is higher than that of patients with the mixed type and the HV type, and the diagnostic confidence patients with the HV type is the lowest. This may be because the HV is thin, or the scanning phase is not accurate thus leading to unclear HV display. Additionally, due to HV occlusion and the formation of intrahepatic collateral vessels, blood vessels may appear unclear.

## Conclusion

In conclusion, spectral CT provides a set of monochromatic energy levels that improved image quality in cases of BCS when combined with ASIR reconstruction technology. A monochromatic energy level of 60 keV in conjunction with 50% ASIR may be the best combination for reducing noise while providing higher overall image quality.
